# High levels of polymorphisms related to raltegravir resistance among raltegravir-naïve individuals in Brazil

**DOI:** 10.1186/1758-2652-13-S4-P135

**Published:** 2010-11-08

**Authors:** N Mantovani Pena, R Gonçalves Azevedo, D Funayama Castro, R Sobhie Diaz, S Vasconcelos Komninakis

**Affiliations:** 1 Federal University of São Paulo, São Paulo, Brazil; 2 Lusiada Fundation of Santos, Santos, Brazil

## Purpose of the study

Raltegravir (RAL) is an HIV-1 integrase strand-transfer inhibitor that has exhibited substantial efficacy and a favorable safety profile in HIV-1 infected patients. The goal of this study was to explore the presence of natural polymorphisms and primary mutations related to RAL resistance among HIV-1 patients failing to multiple antiretroviral agents.

## Methods

25 plasmas from HIV-1 infected patients with HAART failure were studied. Genetic analysis was performed amplifying and sequencing DNA encompassing 288 amino acids of HIV-1 integrase gene. Drug resistance mutations and polymorphisms were examined following Low et al, 2009. Genetic subtypes were analyzed using REGA HIV Subtyping Tool (http://www.bioafrica.net/subtypetool/html/subtypinghiv.html).

## Results

Of the 25 patients, 15 were males and 10 females. All of them are more than 18 years old and 19 patients born in Sao Paulo city. 22 patients were infected by HIV-1 subtype B, 1 by subtype F and 2 by B/F recombinants. No Raltegravir resistance related mutations were observed, however we identified following polymorphisms: V72I (44%), T97A (4%), Q146K (4%), V151I (28%), V201I (52%), T206S (8%), I203M (12%), S230N (4%), M154L (4%), K156N (16%) e K156R (4%). Furthermore, we observed amino acid substitutions at codons 163 in two patients (G163E e G163V) and 138 in one patient (E138N).

## Conclusions

Despite the absence of RAL primary resistance mutations, we found a high frequency of polymorphisms that were related to in vitro reduced susceptibility to RAL. Furthermore, substitutions at codons 163 (G163R) and 138 (E138K) are called secondary mutations, which are capable to restore viral fitness due to the presence of primary mutations. Further studies are needed to determine the importance of these polymorphisms in reducing the genetic barrier to RAL resistance among treated individuals.
